# THE TROUBLE WITH FORGETTING: PROSPECTIVE AND RETROSPECTIVE MEMORY FAILURES AMONG CAREGIVERS

**DOI:** 10.1093/geroni/igae098.2424

**Published:** 2024-12-31

**Authors:** Rachael Turner, Anisha Sharma, Celinda Reese-Melancon

**Affiliations:** Oklahoma State University, Kearney, Nebraska, United States; Oklahoma State University, Stillwater, Oklahoma, United States; Oklahoma State University, Stillwater, Oklahoma, United States

## Abstract

Caregivers serve a critical role in assisting their loved ones, though providing care can be associated with physical and cognitive decline. We investigated disparities in memory lapses and their perceived impact on daily functioning among caregivers and non-caregivers. We focused on lapses in prospective memory (PM; i.e., memory for future actions) and retrospective memory (RM; i.e., remembering information from the past). Participants included respondents of the Midlife in the United States Refresher 1: Daily Diary Project dataset (Ryff & Almeida, 2020). Individuals were identified as caregivers if they indicated assisting a spouse or parent with a disability or health condition throughout the week (n = 75). Self-report questionnaires measured instances of PM and RM forgetting, interference with daily routine resulting from forgetting, how bothered one is by forgetting, positive and negative affect, and time spent on caregiving tasks. Contrary to expectations, caregivers did not differ from non-caregivers on frequency of forgetting PM or RM tasks (ps >.05). Additional analyses investigated relationships between key variables and everyday forgetting and indicated that greater PM or RM forgetting among caregivers was associated with being more bothered by forgetting and more interference with daily routine (ps <.001). Further, negative affect and time spent caregiving significantly predicted caregivers’ PM forgetting with caregivers who reported greater negative affect and more time spent caregiving reporting more instances of PM forgetting. The present study highlights the subjective experiences of caregivers in managing their daily memory tasks.

